# Preclinical Optimization and Safety Studies of a New Lentiviral Gene Therapy for p47^phox^-Deficient Chronic Granulomatous Disease

**DOI:** 10.1089/hum.2020.276

**Published:** 2021-09-23

**Authors:** Andrea Schejtman, Winston Vetharoy, Uimook Choi, Christine Rivat, Narda Theobald, Giuseppa Piras, Diego Leon-Rico, Karen Buckland, Elena Armenteros-Monterroso, Sara Benedetti, Michael T. Ashworth, Michael Rothe, Axel Schambach, Hubert Bobby Gaspar, Elizabeth M. Kang, Harry L. Malech, Adrian J. Thrasher, Giorgia Santilli

**Affiliations:** ^1^ Molecular and Cellular Immunology Unit, UCL Great Ormond Street Institute of Child Health, University College London, London, United Kingdom.; ^2^ Laboratory of Clinical Immunology and Microbiology, National Institute of Allergy and Infectious Diseases, National Institutes of Health, Bethesda, Maryland, USA.; ^3^ Great Ormond Street Hospital for Children, NHS Foundation Trust, London, United Kingdom.; ^4^ Department of Histopathology, Great Ormond Street Hospital for Children, NHS Foundation Trust, London, United Kingdom.; ^5^ Institute of Experimental Hematology, Hannover Medical School, Hannover, Germany.; ^6^ Division of Hematology/Oncology, Boston Children's Hospital, Harvard Medical School, Boston, Massachusetts, USA.; ^7^ Orchard Therapeutics, London, United Kingdom.

**Keywords:** chronic granulomatous disease, genotoxicity, biodistribution, lentiviral gene therapy

## Abstract

Chronic granulomatous disease (CGD) is an inherited blood disorder of phagocytic cells that renders patients susceptible to infections and inflammation. A recent clinical trial of lentiviral gene therapy for the most frequent form of CGD, X-linked, has demonstrated stable correction over time, with no adverse events related to the gene therapy procedure. We have recently developed a parallel lentiviral vector for p47^phox^-deficient CGD (p47^phox^CGD), the second most common form of this disease. Using this vector, we have observed biochemical correction of CGD in a mouse model of the disease. In preparation for clinical trial approval, we have performed standardized preclinical studies following Good Laboratory Practice (GLP) principles, to assess the safety of the gene therapy procedure. We report no evidence of adverse events, including mutagenesis and tumorigenesis, in human hematopoietic stem cells transduced with the lentiviral vector. Biodistribution studies of transduced human CD34^+^ cells indicate that the homing properties or engraftment ability of the stem cells is not negatively affected. CD34^+^ cells derived from a p47^phox^CGD patient were subjected to an optimized transduction protocol and transplanted into immunocompromised mice. After the procedure, patient-derived neutrophils resumed their function, suggesting that gene correction was successful. These studies pave the way to a first-in-man clinical trial of lentiviral gene therapy for the treatment of p47^phox^CGD.

## Introduction

Chronic Granulomatous Disease (CGD) is a genetic disorder of the innate immune system caused by a defective nicotinamide adenine dinucleotide phosphate (NADPH) oxidase complex, the enzyme that drives pathogen clearance through production of reactive oxygen species and regulation of phagosomal pH and ionic content.^1,2^ Around 1:200,000 individuals are born with CGD worldwide and suffer from life-threatening infections and inflammatory complications. There are X-linked and autosomal recessive forms of the disease depending on which subunit of the NADPH enzymatic complex is affected by the genetic mutation. The most common autosomal recessive form is p47^phox^ deficiency that accounts for 25% of CGD cases in Western countries with a higher frequency in Eastern countries where the number of consanguineous marriages is elevated.^3,4^ Although generally less severe than the X-linked form, p47^phox^-deficient CGD (p47^phox^CGD) still causes significant morbidity and mortality mainly due to severe gastrointestinal disorders.^5^ For years, hematopoietic stem cell transplantation^6^ has been the only cure for individuals affected by CGD but the advent of *ex vivo* gene therapy has unveiled new therapeutic options.^7^

We have previously reported on the efficacy of a lentiviral vector designed for the gene therapy of p47^phox^CGD in a mouse model of p47^phox−/−^ upon challenge with the *Salmonella* Typhimurium pathogen, a prominent cause of septicemia in CGD patients.^8^ The lentiviral transfer plasmid used in the study, pCCLCHIM-p47^phox^, contains a myeloid internal promoter that drives expression preferentially in granulocytes mainly affected by CGD.^9^ Similar lentiviral vectors, containing either the *CYBB* or the *ITGB2* transgene are currently used in phase I/II clinical trials for the X-linked form of CGD (X-CGD) and for leukocyte adhesion deficiency type-1 (LAD-I), respectively (trial registry numbers NCT02234934/NCT01855685, NCT03812263/NCT03825783). Both trials have shown promising results in terms of efficacy^10,11^ with no vector-related adverse events (more than 3 years of follow-up for some X-CGD patients). Comprehensive genotoxicity studies had been conducted, before the start of clinical trials, to confirm the safety profile of each vector.^12,13^ Data from those can be used to inform the safety profile of the lentiviral vector that we propose for the gene therapy of p47^phox^CGD (here referred as LV.CHIM-p47), as the regulatory elements are identical.

The present work reports on safety studies for the LV.CHIM-p47 lentiviral vector that were performed following Good Laboratory Practice (GLP) principles. In this study, we show that the lentiviral gene therapy strategy does not alter the output of hematopoietic cells or their engraftment ability *in vivo* and does not cause cancer. When transducing CD34^+^ hematopoietic stem and progenitor cells (CD34^+^ HSPCs) with the lentiviral vector in the presence of transduction enhancers, we could achieve a good rescue of NADPH oxidase activity in CD34^+^-derived human granulocytes.

This work supports the implementation of a phase I/II lentiviral gene therapy trial for p47^phox^CGD.

## Results and Discussion

### Clinical vector

The self-inactivating (SIN) lentiviral transfer plasmid pCCLCHIM-p47^phox^, depicted in Supplementary Fig. S1, is described in Schejtman *et al.*^8^ The vector was produced by the manufacturing department of Indiana University Cell and Gene Therapy Manufacturing facility (IU-CGTM) according to good manufacturing practice (GMP) and its titer was determined on HT29 cells by quantitative polymerase chain reaction (qPCR) as described in Supplementary Data.

### Aim and strategy

The main aim of this study was to test the safety profile of the LV.CHIM-p47 vector. To this purpose we used (1) a well-established *in vitro* immortalization assay (*in vitro* genotoxicity), (2) transplantation experiments using the p47^phox^CGD mouse model (*in vivo* genotoxicity), (3) xenotransplantation experiments using vector-treated CD34^+^ HSPCs (biodistribution). The experiments were carried out with a GMP-comparable batch; p47CGD-17-2-VP-27GC, vector titer 3 × 10^8^ infectious genomic per milliliter (IG/mL).

The second aim of this study was to test the clinical vector (p47CGD-18-2-VP-27, vector titre 6.3 × 10^9^ IG/mL) on patient's cells using the combination of poloxamer F108^14^ (LentiBOOST) and Protamine Sulfate as transduction enhancers, following protocols already established in our laboratory.^15^

### Summary of data

#### Genotoxicity

##### In vitro

The *in vitro* immortalization (IVIM) assay performed on murine Lineage-negative cells (Lin^−^) was used to assess the genotoxicity of the LV.CHIM-p47 vector. This assay relies on the induction of a survival advantage by insertional activation of cellular proto-oncogenes, which becomes evident only when primary murine hematopoietic cells are cultured under differentiating cytokine conditions and plated in a limiting dilution.^16^ A detailed description of the IVIM assay can be found in Supplementary Materials and Methods (in Supplementary Data). A number of positive and negative controls were included in the assay. As positive controls, we used the RSF91.GFPgPRE gamma-retroviral long terminal repeat (LTR)-driven vector (RSF91) with a documented potential to induce *in vitro* immortalization by insertional mutagenesis,^17^ and historical data (metadata) on the SIN-lentiviral vector RRL.PPT.SF.eGFP.pre (MA-Lv-SF), in which the eGFP transgene expression is under the control of the SFFV U3 as an internal promoter. As negative controls, we used samples for which no retroviral transduction was performed to determine the rate of immortalized cells due to spontaneous mutations (Mock), together with historical data (MA-Mock). Briefly, Lin^−^ cells were exposed to two consecutive rounds of infection with the gene transfer vectors achieving a cumulative multiplicity of infection (MOI) of 400 for the LV.CHIM-p47 vector (LV.p47) and a cumulative MOI of 60 for the RSF91 vector. LV.p47 samples had a high average vector copy number per cell (VCN) of 7.56 ± 1.74 and were in the same range as the positive control RSF91 with a VCN of 7.50 ± 0.73 (Supplementary Table S1).

Despite high VCN values, our candidate vector showed proliferation rates within the expected ranges of previously measured Mock controls (MA-Mock), as did the Mock and RSF91 controls (Supplementary Fig. S2). This argues against a general toxic effect of the vector supernatant or the transgene. Three days after a split, on day 11 of the assay, LV.p47 samples showed a slight but still significant reduction in proliferation compared with MA-Mock. However, the cultures recovered steadily after day 13. After the replating step by limiting dilution on day 15, the cells were cultured for 2 weeks before screening the wells for clonal outgrowth based on four categories: C1, C2, C3, and C4 (see Supplementary Materials and Methods for more details), C1 being the most obvious case of immortalized cultures. Table 1 shows the number of positive wells, either microscopically identified as C1/2 or by the 3-(4,5-dimethylthiazol-2-yl)-2,5-diphenyltetrazolium bromide (MTT)-assay in dubious cases.

**Table 1.** *Microscopic score and MTT-assay score of emerging clones in the* in vitro *immortalization assay*

Vector	c.MOI	VCN (Day 4)	Microscopic Score (Cat1/2)	MTT (Score)	MTT-RF
MOCK	—	—	1	0	0.00E+00
MOCK	—	—	0	0	0.00E+00
MOCK	—	—	0	0	0.00E+00
MOCK	—	—	0	0	0.00E+00
MOCK	—	—	0	0	0.00E+00
MOCK	—	—	0	0	0.00E+00
MOCK	—	—	7	1	1.05E−04
MOCK	—	—	2	0	0.00E+00
MOCK	—	—	6	0	0.00E+00
RSF91	60	6.8	25	22	2.60E−03
RSF91	60	7.31	36	52	7.81E−03
RSF91	60	6.66	19	35	4.52E−03
RSF91	60	7.02	14	18	2.07E−03
RSF91	60	7.78	3	3	3.17E−04
RSF91	60	8.78	13	4	4.26E−04
RSF91	60	7.29	4	4	4.26E−04
RSF91	60	8.52	0	51	7.58E−03
RSF91	60	7.38	4	3	3.17E−04
LV-p47	400	7.8	0	0	0.00E+00
LV-p47	400	7.68	0	0	0.00E+00
LV-p47	400	7.61	0	0	0.00E+00
LV-p47	400	8.46	0	0	0.00E+00
LV-p47	400	6.71	1	0	0.00E+00
LV-p47	400	7.08	0	0	0.00E+00
LV-p47	400	7.06	0	0	0.00E+00
LV-p47	400	6.19	1	0	0.00E+00
LV-p47	400	5.84	1	0	0.00E+00
LV-p47	400	9.68	0	0	0.00E+00
LV-p47	400	6.89	0	0	0.00E+00
LV-p47	400	9.86	0	0	0.00E+00
LV-p47	400	11.51	3	0	0.00E+00
LV-p47	400	6.16	2	0	0.00E+00
LV-p47	400	4.8	0	0	0.00E+00

MTT, 3-(4,5-dimethylthiazol-2-yl)-2,5-diphenyltetrazolium bromide; MTT-RF, MTT-replating frequency; VCN, average vector copy number per cell.

Mock control plates contained wells of microscopic category C2 to C4. No C1 clones were detected for Mock.

**Figure 1.**
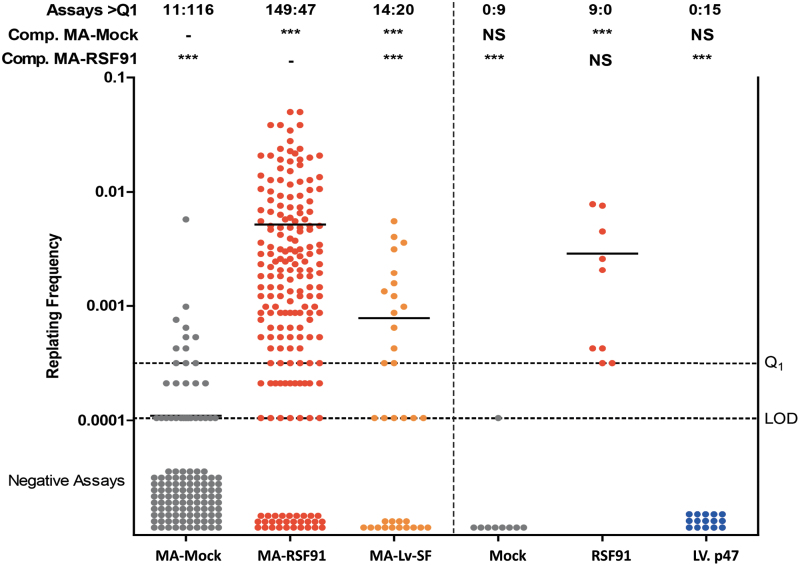
IVIM assay determining the risk of insertional mutagenesis. RF of the control samples Mock or RSF91 and the test vector LV.CHIM-p47 (LV. p47) in comparison to data of a meta-analysis for control samples (MA-Mock, MA-RSF91, MA-Lv-SF). For clarity, the historical data are separated from the experimental ones with a *vertical dotted line*. The data points below the LOD (plates with no wells above the MTT-threshold) were manually inserted into the graph (due to the logarithmic scale of the y-axis). Above the graph, the ratio of positive (*left number*) and negative plates (*right number*) according to the MTT-assay are shown. Differences in the incidence of positive and negative assays relative to MA-Mock or MA-RSF91 were analyzed by Fisher's exact test with Benjamini**–**Hochberg correction (***p < 0.001; NS, not significant). Bars indicate the mean RF. IVIM, *in vitro* immortalization; LOD, limit of detection; MTT, 3-(4,5-dimethylthiazol-2-yl)-2,5-diphenyltetrazolium bromide (MTT) reduction assay; RF, replating frequency. Color images are available online.

The positive control RSF91 showed C1 wells, in eight out of nine plates. After the sensitive and more objective MTT-analysis, all nine positive control vector plates were above the quantification threshold and could be classified as proper clones. The incidence of plates with insertional mutants and the mean replating frequency (2.9 × 10^−3^) was statistically not different from the meta-data of RSF91 (Fig. 1). For the LV.CHIM-p47 vector, no C1 wells were detectable, and only 5 out of 15 plates were microscopically scored as C2. In the MTT-assay, LV.p47 plates were all below the quantification threshold and so could not be classified as true clones. From this set of experiments we can conclude that the LV.CHIM-p47 has a significantly lower genotoxic potential compared with RSF91 gamma retroviral vector and its clonogenic potential is indistinguishable from that of nontransduced cells in the IVIM assay, even at high copy numbers per cell.

##### In vivo

**Figure 2.**
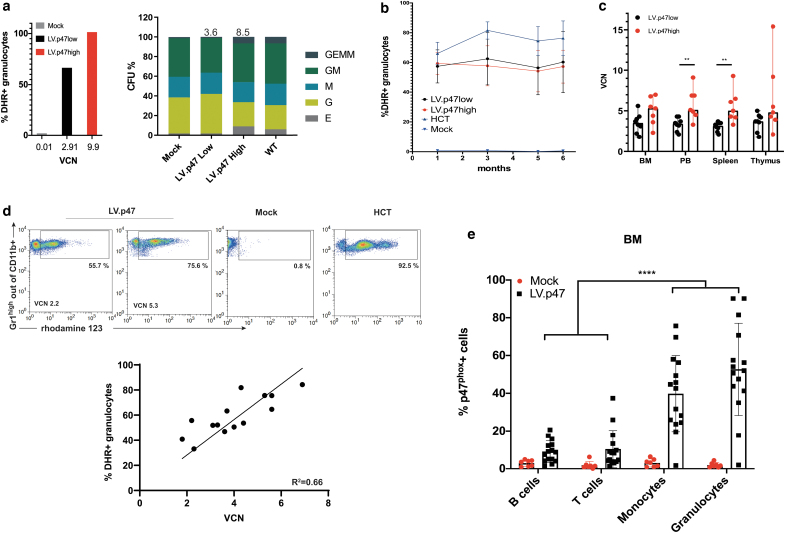
Biochemical correction of p47^phox−/−^ mice by lentiviral gene therapy. p47^phox−/−^ Lin^−^ cells were transduced with the LV.CHIM-p47 vector at a MOI of 50 (LV.p47low) or 300 (LV.p47high) and transplanted into lethally irradiated p47^phox−/−^-recipent mice (*n* = 8). As control we used p47^phox−/−^ mice transplanted with untransduced p47^phox−/−^ (Mock; *n* = 8) or wild-type (HCT; *n* = 8) Lin^−^ cells and untreated p47^phox−/−^ (KO; *n* = 4) or C57BL/6 (WT; *n* = 6) mice. Mice were analyzed up to 6 months after transplantation. **(a)** VCN and percentage of functional (% of DHR positive) granulocytes in lentivirally transduced p47^phox−/−^ Lin^−^ cells upon granulocytic differentiation in liquid cultures (*left panel*). CFUs and VCN in pooled colonies (*right panel*). **(b)** DHR over time in peripheral blood granulocytes of gene therapy-treated mice (LV.p47low and LV.p47high) and of mice in the HCT or Mock groups. **(c)** VCN in different hematopoietic organs of mice transplanted with LV.p47low (*black*) or LV.p47 high (*red*) transduced cells. Data are presented as median and range; Mann–Whitney test, **p < 0.01. **(d)** Representative DHR plot in granulocytes from the bone marrow of LV.p47 (low and high), Mock, and HCT groups (*upper panel*). The *lower panel* shows the correlation between percentage of DHR-positive granulocytes found in the bone marrow of gene therapy-treated p47^phox−/−^ mice and vector copy number (1.5< VCN <7). *R*^2^ = 0.66, squared Perason's correlation coefficient, *p* = 0.0004. **(e)** p47^phox^ expression in B cells (B220^+^), T cells (CD3^+^), monocytes (CD11b^+^/Gr1low), granulocytes (CD11b^+^/Gr1 high) in the bone marrow of gene therapy-treated mice (*n* = 15). Data are mean ± SD; two-way ANOVA with Tukey's multiple comparison, ****p < 0.001. ANOVA, analysis of variance; DHR, dihydrorhodamine; HCT, hematopoietic cell transplantation group; KO, knock out group; MOI, multiplicity of infection; SD, standard deviation; VCN, average vector copy number per cell. Color images are available online.

We performed an *in vivo* genotoxicity study using the p47^phox−/−^ murine model. For this set of experiments, we transduced p47^phox−/−^ lineage-negative cells with the LV.CHIM-p47 vector at low, 50 (LV.p47low) and high, 300 (LV.p47high) MOI and transplanted them into lethally irradiated p47^phox−/−^-recipient mice (*n* = 8 for each MOI). To avoid potential toxicity, we used a cumulative MOI of 300, with two sequential transduction of MOI 150 each. Part of the cells were cultured in neutrophil differentiation medium to evaluate the recovery of ROS production by a dihydrorhodamine (DHR) assay and plated in semisolid media to monitor the differentiation potential alongside with vector copies (Fig. 2a).

Mice transplanted with untransduced p47^phox−/−^ (Mock group; *n* = 8) or wild-type (HCT, hematopoietic cell transplantation group; *n* = 8) Lin^−^ cells were used as negative and positive control, respectively. We also included a cohort of C57BL/6 (WT group; *n* = 6) and p47^phox−/−^ (KO group; *n* = 4) animals to rule out any toxicity due to the gene therapy procedure itself.

Following transplantation, mice were monitored for up to 26 weeks with regular tail vein bleeds to check for the persistence of corrected cells over time (Fig. 2b and Supplementary Table S2). One mouse in the HCT group had to be sacrificed shortly after the gene therapy procedure due to engraftment failure. One mouse in the Mock group died during a tail vein bleed. One mouse in the LV. p47high group had to be sacrificed 4 months after transplantation following the development of a nasal granuloma, which has previously been shown to be a recurrent spontaneous infection in this mouse model.^18,19^ Macroscopic examination of internal organs did not reveal any anomaly. Of note, this mouse (#10) had no gene therapy-corrected cells in the blood at any time point as shown by the lack of DHR positivity and VCN, suggesting that the graft was not successful (Supplementary Table S2). At the end of the experiment (6 months after transplantation), we analyzed the biochemical reconstitution of gene therapy-treated mice and the number of vector integrants. The analysis of VCN revealed that the majority of gene therapy-treated mice had between 3 and 6 vector copies in hematopoietic organs (Fig. 2c), confirming multiple integration events, a finding that underpins the validity of this genotoxicity study. We also found a good correlation (*R*^2^ = 0.66) between vector copies and DHR positivity in the bone marrow of gene therapy-treated mice, with around 2 vector copies needed to achieve more than 30% functional neutrophils (Fig. 2c). Finally, we confirmed in this setting as previously shown^8^ that the LV.CHIM-p47 lentiviral vector drives p47^phox^ expression preferentially in granulocytes and monocytes (Fig. 2d).

**Figure 3.**
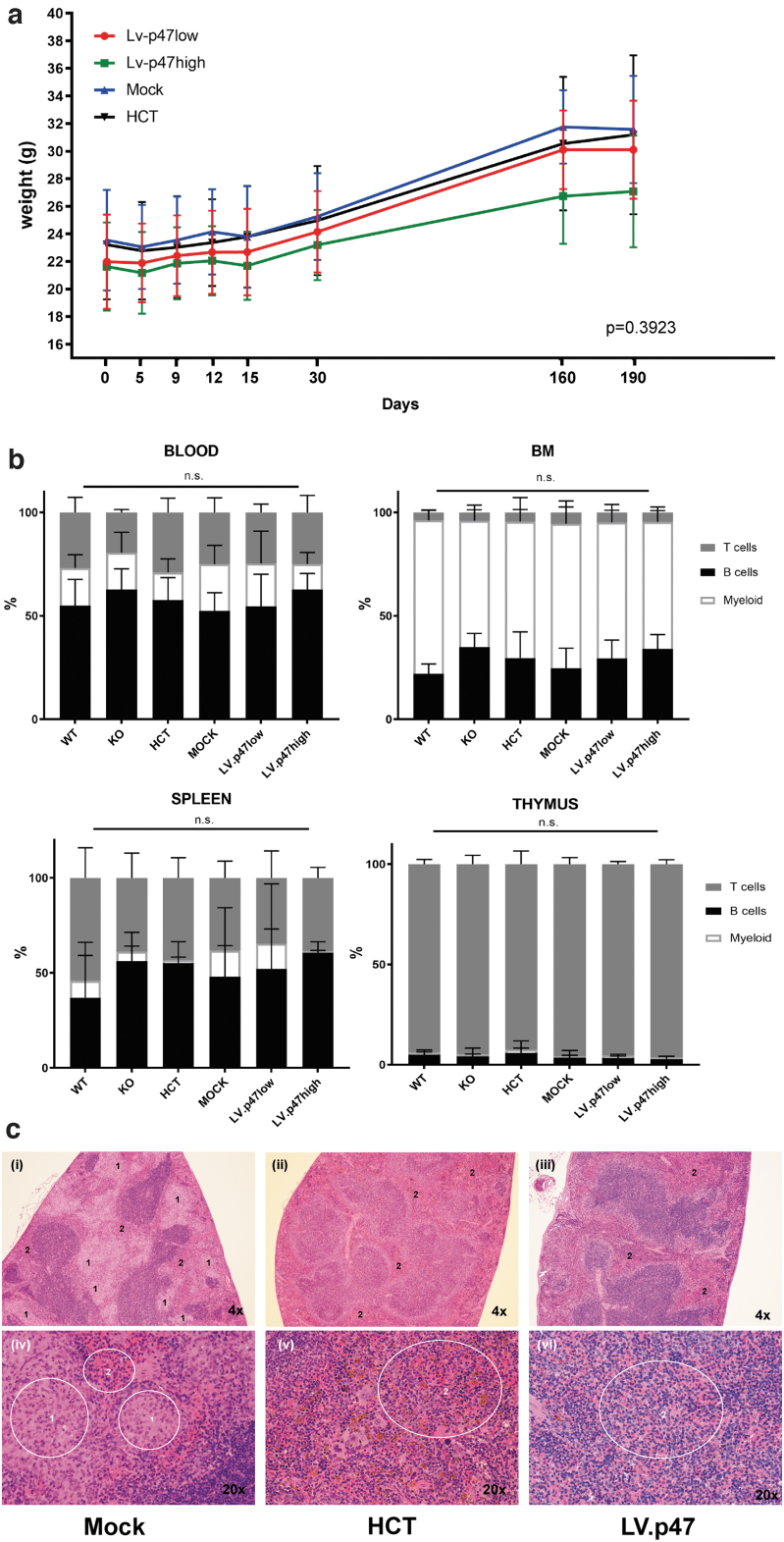
*In vivo* genotoxicity: body weight, hematological and histopathological analysis of transplanted mice. **(a)** Monitoring of body weight (g) over time in mice from the LV. p47 low (*n* = 8), LV. p47 high (*n* = 7), Mock (*n* = 7), and HCT (*n* = 7) groups. Simple linear regression analysis showing no difference between the slopes (*p* = 0.3923). **(b)** Percentage of T cells (CD3^+^), B cells (B220^+^), and myeloid cells (CD11b^+^) in PB, BM (KO, *n* = 4; WT, *n* = 6; Mock, *n* = 7; HCT, *n* = 7; LV.p47low, *n* = 8; LV.p47high, *n* = 7), and spleen or thymus (KO, *n* = 4; WT, *n* = 3 or *n* = 6; Mock, *n* = 7; HCT, *n* = 6; LV.p47low, *n* = 8; LV.p47high, *n* = 6 or *n* = 5). One hundred percent is given by the total percentage of B, T, and myeloid cells. Data are mean ± SD; two-way ANOVA followed by Tukey's multiple comparison, ns, not significant. **(c)** Hematoxylin and eosin staining of spleen sections from representative animals belonging to the Mock (“i”, “iv”), HCT (“ii”, “v”), and LV.p47 (“iii”, “vi”) groups at low (*upper panels*) and high (*lower panels*) magnification. Areas labeled as “2” show normal *red* pulp of the spleen, whereas areas labeled as “1” indicate macrophage infiltration. BM, bone marrow. Color images are available online.

In terms of general health, all animals increased body weight over time and similar growth trajectories were exhibited among all experimental groups (Fig. 3a). Flow cytometric analysis of the B, T, and myeloid compartment of peripheral blood, bone marrow, spleen, and thymus (Fig. 3b and Supplementary Fig. S3) showed no difference in hematopoiesis among the experimental groups. The spleen of one mouse belonging to the LV.p47low group (Supplementary Fig. S3) exhibited an elevated number of myeloid cells by flow cytometry. However, that sample contained >90% dead events, a confounding factor that could have impacted the technical validity of the data. Of note, the same mouse had normal lineage distributions in the other organs, normal blood parameters (Supplementary Fig. S4a–d), normal spleen weight (Supplementary Fig. S4e), and normal histology as discussed below. Hematological analysis showed that hemoglobin levels (HGB) and total count of erythrocytes (RBCs), white blood cells (WBCs), or circulating platelets (PLT) were similar between all groups.

Histopathological analysis of hematopoietic organs did not reveal any evidence of hematopoietic malignancy (Supplementary Data). The thymus had normal cellular density of lymphocytes in most animals and minimal-to-mild hypercellularity of the bone marrow was observed in all five experimental groups with no obvious difference between groups with regard to bone marrow histology. Splenic architecture and cellularity was normal in the majority of animals. Of note, four mice, two in the KO group (50%) and two in the Mock group (29%) showed macrophage infiltration either of the bone marrow or of the splenic red pulp. Infiltrating macrophages were often clustered together forming multifocal granuloma-like lesions, indicative of an inflammatory state.^20^ Granuloma in the splenic red pulp of a representative mouse belonging to the Mock group (i, iv), characterized by macrophages with abundant pale cytoplasm compressing surrounding cells, is shown in Fig. 3c. In contrast, in the HCT (ii, v) and LV.p47 (iii, vi) groups the splenic red pulp showed an even dispersion of multiple cell types, with no mechanical tissue displacement, suggesting a rescue of the inflammatory phenotype by gene therapy.

From this set of experiments we can conclude that transduction with the LV.CHIM-p47 lentiviral vector does not readily induce tumorigenesis. It is worth highlighting that CGD mice suffer from underlying pre-existing inflammation, a condition which, according to a recent report, can increase tumor incidence in gene therapy settings.^21^ Notably, our *in vivo* study was performed on the p47^phox^CGD mouse model.

#### Biodistribution

To conclude the safety analysis of our lentiviral gene therapy strategy, we evaluated the effects of lentiviral transduction on the engraftment and repopulation ability of human CD34^+^ cells from mobilized blood, which is the most common starting material for clinical gene therapy. Indeed, transduction of hematopoietic stem cells in the context of gene therapy results in the expression of the therapeutic gene in progenitor cells and may alter their innate homing and engraftment properties or their ability to differentiate into myeloid and lymphoid lineages. Therefore, we performed a biodistribution study using human CD34^+^ cells that were transplanted into immunodeficient non-obese diabetic (NOD)-SCID Il2rg^−/−^ (NSG) mice to evaluate any potential accumulation of gene-transduced cells in nonhematopoietic organs (Supplementary Data), as already described in Carriglio *et al.*^22^ Following the protocol of the current gene therapy trial for X-CGD,^10^ we transduced CD34^+^ cells with the LV.CHIM-p47 vector using a cumulative MOI of 200 with two consecutive hits of MOI 100, and transplanted the cells into 10 female and 10 male sublethally irradiated NSG mice (LV.p47). Twenty NSG mice (10 females, 10 males) transplanted with untransduced CD34^+^ cells were used as control for the experiment (UN). Some of the mice experienced a weight loss of ∼10% soon after bone marrow conditioning and cell transplantation, but overall all of the mice gained weight overtime and survived until the end of the experiment, which suggests that neither the conditioning nor the gene therapy treatment are toxic in this setting (Supplementary Fig. S5a). At 3 months posttransplantation, mice were sacrificed and the hematopoietic (peripheral blood, bone marrow, spleen, and thymus) and nonhematopoietic (kidney, muscle, lung, heart, brain, liver, and gonads) organs were harvested. We evaluated the human cell engraftment in hematopoietic organs by flow cytometry staining for hCD45, and also by quantifying the human albumin gene through droplet digital PCR (ddPCR). For analyzing the engraftment in nonhematopoietic organs, we merely used ddPCR because of the very low levels of engraftment expected in those tissues.

The hCD45 staining of bone marrow showed good levels of human cell engraftment in both UN and LV.p47 mice, with no significant difference among the groups (Table 2 and Supplementary Fig. S5b). Vector copies ranging between 1.0 and 2.8 were found in the bone marrow (BM) of experimental mice with no difference between sex (Table 2 and Supplementary Fig. S5c). In both experimental groups, we found a high percentage of CD19^+^ B cells and a smaller percentage of CD13^+^ myeloid cells as expected in NSG mice that predominantly sustain the development of B cells^23^ (Supplementary Fig. S5d). The UN and LV.p47 groups had similar levels of human cell engraftment and lineage representation in peripheral blood, spleen, and thymus (data not shown) demonstrating once more that the vector does not have a negative impact on the engraftment capacity of gene therapy-treated cells. As expected, engraftment of human cells into the hematopoietic organs (blood, bone marrow, spleen, and thymus) of the recipient mice was much higher than in nonhematopoietic organs (Table 3). The average engraftment in the bone marrow was ∼50%, whereas engraftment of human cells in all nonhematopoietic organs was between 0.5% and 3.6% in liver, lung, muscle, and kidney and lower than 0.5% in brain, gonads, and heart, as assessed by ddPCR. We did not detect any difference in the distribution of human cells in nonhematopoietic tissues between the LV.p47 and UN groups. Importantly, vector copies were found only in the presence of the human albumin genome and in a similar range as in target organs, suggesting that the vector is present only in human cells and that vector-bearing cells do not accumulate in nontarget organs.

**Table 2.** Human cell engraftment and average vector copy number per human cell in hematopoietic organs of NSG mice transplanted with untransduced (UN) or LV.CHIM-p47 transduced (LV. p47) CD34^+^ cells

Group	Sex	PB			BM			Spleen			Thymus		
		%CD45	%hAlb	VCN	%CD45	%hAlb	VCN	%CD45	%hAlb	VCN	%CD45	%hAlb	VCN
UN	Male	4.38 ± 1.86	9.68 ± 11.50	N/A	26 ± 8	37 ± 13	N/A	25 ± 17	39 ± 15	N/A	5.75 ± 6.87	0.87 ± 0.56	N/A
	Female	4.44 ± 3.80	4.17 ± 5.02^a^	N/A	33 ± 9	54 ± 10	N/A	19 ± 17	46 ± 11	N/A	3.14 ± 2.99	0.68 ± 0.46	N/A
LV.p47	Male	5.11 ± 3.42	7.23 ± 12.51	1.86 ± 0.72	32 ± 15	40 ± 18	1.89 ± 0.49	27 ± 18	40 ± 12	1.65 ± 0.28	3.42 ± 1.84	1.12 ± 0.89	2.15 ± 0.39
	Female	3.91 ± 1.87	7.35 ± 8.31	2.34 ± 0.80	41 ± 7	56 ± 8	1.85 ± 0.47	27 ± 21	36 ± 11	1.70 ± 0.20	2.25 ± 0.45	1.20 ± 0.69	2.05 ± 0.55

Percentage of human cells detected by flow cytometry (hCD45^+^) and ddPCR (hAlb: hAlb/hAlb+mTitin) in the peripheral blood and lymphoid organs with respective average vector copy number per human cell; see Supplementary Materials and Methods for more information. Results are shown as mean ± SD calculated on numerical values >LOQ from animals showing successful engraftment (defined as ≥1% human CD45^+^ cells in BM at week 11). Statistical analysis: one-way ANOVA followed by Tukey's multicomparison, ns = not significant between UN and LV. p47 groups in all the organs.

^a^ Three samples showing an engraftment of 86% and 82% and 61% according to ddPCR were removed (outliers were detected by a Grubb's test considering a totality of 36 samples in PB).

ANOVA, analysis of variance; BM, bone marrow; ddPCR, droplet digital polymerase chain reaction; LOQ, limit of quantification; N/A, not available; NSG, immunodeficient non-obese diabetic (NOD)-SCID Il2rg^−/−^; PB, peripheral blood; SD, standard deviation.

**Table 3.** Human cell engraftment and average vector copy number per human cell in nonhematopoietic organs of NSG mice transplanted with untransduced (UN) or LV.CHIM-p47 transduced (LV. p47) CD34^+^ cells

Group	Sex	Heart		Kidney		Lung		Liver		Muscle		Brain		Gonads	
		%hAlb	VCN	%hAlb	VCN	%hAlb	VCN	%hAlb	VCN	%hAlb	VCN	%hAlb	VCN	%hAlb	VCN
UN	Male	0.05 ± 0.04	N/A	0.99 ± 0.31	N/A	3.13 ± 1.08	N/A	0.74 ± 0.31	N/A	0.72 ± 0.57	N/A	0.02 ± 0.06	N/A	0.02 ± 0.02	N/A
	Female	0.05 ± 0.02	N/A	0.59 ± 0.22	N/A	3.10 ± 0.38	N/A	1.10 ± 0.20	N/A	2.01 ± 3.74	N/A	0.04 ± 0.05	N/A	0.25 ± 0.17	N/A
LV.p47	Male	0.06 ± 0.03	<LOQ	1.22 ± 0.30	1.91 ± 0.60	3.54 ± 2.06	1.81 ± 0.64	0.65 ± 0.38	2.30 ± 0.20	0.53 ± 0.85	1.44 ± 0.89	0.04 ± 0.04	<LOQ	0.02 ± 0.01	N/A
	Female	0.07 ± 0.02	<LOQ	0.53 ± 0.19	1.86 ± 0.26	2.99 ± 1.20	1.96 ± 0.30	1.11 ± 0.44	2.64 ± 1.15	1.82 ± 1.15	2.14 ± 3.99	0.02 ± 0.01	<LOQ	0.19 ± 0.07	2.5^a^

Percentage of human cells detected by ddPCR (hAlb: hAlbumin/hAlbumin+mTitin) in nontarget organs with respective VCN; see Supplementary Materials and Methods for more information. Results are given as mean ± SD calculated on numerical values >LOQ from animals showing successful engraftment (defined as ≥1% human CD45^+^ cells in BM at week 11). Statistical analysis: one-way ANOVA followed by Tukey's multicomparison. n.s. between UN and LV.p47 groups in all the organs.

^a^ Only one animal.

#### Clinical protocol for the gene therapy of human p47^phox^CGD CD34^+^ cells

**Figure 4.**
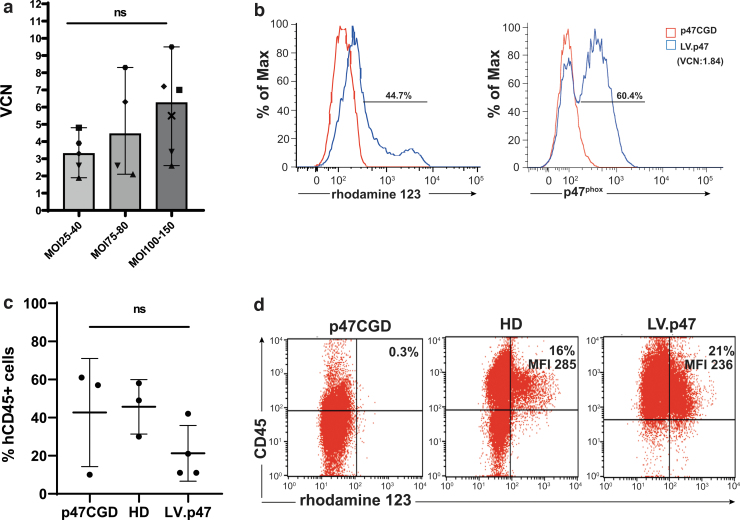
Vector performance on CD34^+^ cells from a healthy donor and from a p47^phox^CGD patient. **(a)** CD34^+^ cells from six healthy donors (each visualized by a different symbol) were transduced with two separate batches of the clinical grade LV.CHIM-p47 vector (p47CGD 18-2-VP-27 and p47CGD 19-I-VM-08; titers of 6.3 × 10^9^ IG/mL and 5.9 × 10^9^ IG/mL, respectively) at different MOI in the presence of 1 mg/mL of LentiBOOST and 4 μg/mL Protamine Sulfate. The x axis shows the range of MOI used and the y axis the VCN. Data are presented as median and range; one-way ANOVA with Tukey's multiple comparison, ns, not significant. **(b)** p47^phox^CGD cells were transduced with the clinical vector (p47CGD 18-2-VP-27) at MOI 130 in the presence of 1 mg/mL of LentiBOOST and 4 μg/mL of Protamine Sulfate, resulting in VCN of 1.84 (shown in *brackets*). DHR activity (*left panel*) and p47^phox^ expression (*right panel*) were assessed by flow cytometry 14 days after culturing CD34^+^ cells into myeloid differentiation medium (with 50 ng/mL of human GCSF). **(c)** Transduced p47^phox^CGD cells (LV. p47) were transplanted into NSG mice (*n* = 4) along with nontransduced p47^phox^CGD cells (p47CGD; *n* = 3) and normal donor CD34^+^ cells (HD; *n* = 3). Human cell engraftment (%CD45^+^ cells) was calculated in bone marrow 16 weeks after transplantation. Data are mean ± SD; one-way ANOVA with Tukey's multiple comparison, ns, not significant. **(d)** DHR activity in CD34^+^-derived myeloid cells (out of FSC high/CD45^+^ cells) from pools of LV.p47, p47CGD, and HD mice. CGD, chronic granulomatous disease; FSC, forward scatter; GCSF, granulocyte colony-stimulating factor; HD, healthy donor; NSG, immunodeficient non-obese diabetic (NOD)-SCID Il2rg^−/−^. Color images are available online.

A bottleneck for any gene therapy clinical trial is the cost and availability of large quantities of “GMP-grade” lentiviral vectors. While we were performing preclinical studies, several reports highlighted the importance of transduction enhancers to reduce vector quantities required for transduction, minimizing the time for *ex vivo* manipulation of CD34^+^ cells.^15,24^ We used GMP-grade clinical vector in combination with 1 mg/mL LentiBOOST and 4 μg/mL Protamine Sulfate to transduce CD34^+^ cells isolated from healthy donors. To combine experiments using different MOIs of the LV.CHIM-p47 vector (peformed in different laboratories), we plotted vector copy numbers against three ranges of MOI, low (MOI 25–40), medium (MOI 75–80), and high (MOI 100–150) (Fig. 4a). As expected, the median of vector copies integrated in CD34^+^ cells using the high MOI range was higher than that obtained with the low range, but the difference was not statistically significant due to the large variability observed in the samples. Some variability between donors has already been reported by others possibly due to different levels of viral restriction factors (*e.g.*, IFITM3) in CD34^+^ cells.^25^

Based on our observations in animal models,^8^ VCN between 2 and 3 resulted in >30% functional neutrophils. Of note, transduction of CD34^+^ HSPCs using the clinical grade LV.CHIM-p47 vector, resulted in two or more lentiviral copies per cell with all ranges of MOI in the presence of transduction enhancers. A recent study conducted on carriers of X-linked CGD showed that 20% functional neutrophils are sufficient to protect against life-threatening infections,^26^ although lower levels also have significant effects.^27^

When the high MOI protocol (MOI 130) was applied to p47^phox^CGD CD34^+^ cells, we achieved a VCN of 1.84 with 60.4% of the cells positive for p47^phox^ and 44.7% of the cells functionally corrected after 14 days of *in vitro* culture (Fig. 4b). The rather low vector copy numbers observed in the CGD samples could be partlially explained by the freezing and thawing of the vector (this was done to preserve vials of the clinical batch). Transduced cells (LV.p47) were able to engraft busulfan-conditioned NSG mice and an average of 5.8 ± 0.7 VCN was found in the bone marrow of gene therapy-treated animals, 16 weeks after transplantation. Due to the high variability in the percentage of hCD45^+^ cells found in the LV.p47 group (*n* = 4) and in mice transplanted with naive p47^phox^CGD (p47CGD, *n* = 3) or healthy donor (HD, *n* = 3) cells, we could not draw any conclusion on the effects of lentiviral transduction on human cell engraftment in this experiment (Fig. 4c). CD34^+^ cells were isolated from pooled bone marrows of NSG mice from each group (LV.p47, p47CGD, HD) and cultured in differentiation media for 3 weeks to assess the percentage of functional neutrophils. We found not only a similar percentage of functional neutrophils between the LV.p47 and HD samples but also similar levels of NADPH oxidase activity as suggested by the values of mean fluoresce intensity in each DHR plot (Fig. 4d).

## Conclusions

Early gene therapy trials for the X-linked form of CGD have highlighted the safety issues concerning the use of gamma retroviral vectors, which could cause insertional mutagensis and clonal expansion.^28,29^

This study shows that the LV.CHIM-p47 vector has an improved safety profile compared with SIN lentiviral vectors with viral promoters and to LTR-driven gamma-retroviral vectors as extrapolated by historical data. In transplantation experiments using the p47^phox−/−^ mouse model, there was no evidence of skewed hematopoiesis related to gene therapy for the observation period (6 months). Taking into consideration our recently published study^8^ together with the current work, a total of 40 p47^phox−/−^ mice were treated with lentiviral gene therapy (primary and secondary transplants) and observed for more than 4 months. In the former study (which was performed with a different preparation of the vector than that used in this study) one mouse had to be sacrificed at 3 months after transplantation due to the occurrence of B cell leukemia as per histopathological analysis of bone marrow and overrepresentation of cells bearing the B220 marker in peripheral blood and BM (data not shown). No tumors were reported in mice transplanted with mock transduced p47^phox−/−^ (0/18) or wild-type (0/19) cells. To evaluate whether the incidence of tumor in the gene therapy group (1/40) was more pronounced than that expected by chance, we performed a chi-square (and Fisher' s exact) test for trend and found a *p*-value of 0.3788, indicating that there is no evidence for vector or procedure-related causality.

In xenotranplantation experiments, we have also shown that the integration of the lentiviral vector in human CD34^+^ cells does not alter the engraftment or the differentiation ability of HSPCs nor the biodistribution of progeny hematopoietic cells. The clinical protocol, using transduction enhancers, resulted in a VCN higher than 2 in almost all the samples analyzed, regardless of the MOI used (from 25 to 150), and in good rescue of NADPH oxidase function when the vector was tested in p47^phox^CGD cells.

In conclusion, in this study, we report that our lentiviral gene therapy protocol is ready for translation into the clinic.

## Supplementary Material

Supplemental data

Supplemental data

Supplemental data

Supplemental data

Supplemental data

Supplemental data

Supplemental data

Supplemental data
