# Supplementary material for: Preclinical Optimization and Safety Studies of a New Lentiviral Gene Therapy for p47phox-Deficient Chronic Granulomatous Disease
Source: Hum Gene Ther. 2021 Sep 23;32(17-18):949–58. doi: 10.1089/hum.2020.276 (PMC8575060; doi:10.1089/hum.2020.276)
Supplement: Supplemental data [file Supp_FigS1.pdf]

Supplementary Figure 1

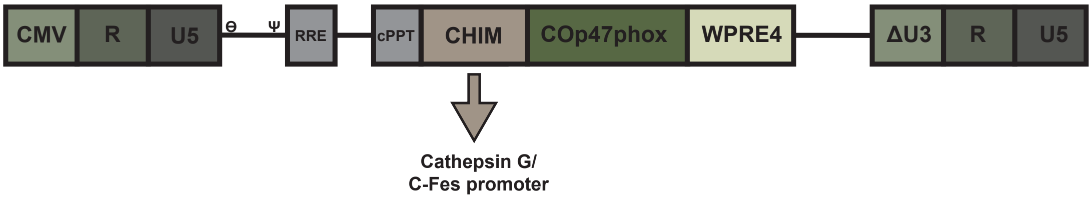

Supplementary Figure 1. Schematic representation of the pCCLCHIM-p47^{phox} lentiviral transfer plasmid [8]. CMV, cytomegalovirus promoter; R and U5 components of HIV-1 LTR; Ψ, HIV-1 packaging signal; RRE, rev responsive element; cPPT, central polypurine tract; CHIM, Cathepsin G/C-Fes promoter; COp47phox, codon optimised *NCF1* coding sequence; WPRE4, woodchuck-hepatitis virus post-transcriptional regulatory element mut 4; ΔU3 RU5, self-inactivating 3' LTR.
