# Supplementary material for: Preclinical Optimization and Safety Studies of a New Lentiviral Gene Therapy for p47phox-Deficient Chronic Granulomatous Disease
Source: Hum Gene Ther. 2021 Sep 23;32(17-18):949–58. doi: 10.1089/hum.2020.276 (PMC8575060; doi:10.1089/hum.2020.276)
Supplement: Supplemental data [file Supp_DataS1.docx]

**SUPPLEMENTARY MATERIAL**

**Materials and Methods**

**Viral titration**

Titration of the LV.CHIM-p47 vector was performed on the human colorectal adenocarcinoma cell line HT-29, using serial dilutions of the viral preparation. Briefly, 24 hrs after seeding 1x10^5^ HT-29 cells, serial dilutions of the vector were added to the wells (1/5,000; 1/50,000; 1/500,000). The media was replaced 20hrs post-transduction, and the cells harvested 48 hrs after media replacement. The titre of the lentiviral vector was determined by qPCR alongside positive control samples with a known titre. The titre is defined as infectious genomes per millilitre (IG/ml). qPCR reactions were set up in triplicates, adding per sample 12.5 µl of Absolute qPCR ROX mix (Thermo Fisher, Carlsbad, CA), 0.225µl of Forward and Reverse of each primer (100 µM)(housekeeping gene and gene of interest), 0.05µl of each probe (100 µM), 5µl of DNA template and 6.5 µl of DNAse free water. Reactions were performed in CFX96 Touch™ Real Time PCR Detection system (BioRad), with denaturation at 95°C for 15 minutes, followed by 40 cycles 15sec at 95°C, 30 sec at 55.6°C and 60sec at 72°C.

For the viral titration in human cells, we used the following primers/probes for HIV: HIV-psi forward 5’_CAGGACTCGGCTTGCTGAAG_3’ and reverse 5’_TCCCCCGCTTAATACTGACG_3’; the HIV-psi probe sequence was 5’_FAM_CGCACGGCAGGCGAGG_TAMRA_3’ and the following primers/probe for human albumin: hAlbumin forward 5’_GCTGCTATCTCTTGTGGGCTGT_3’ and reverse 5’_ACTCATGGGAGCTGCTGGTTC_3’; the hAlbumin probe sequence was 5’_VIC_CCTGTCATGCCCACACAAATCTCTCC_TAMRA_3’.

Analysis of the data was made using CFX Maestro™ Software. Titre values are given by the formula: VCN X the number of cells seeded X dilution factor.

**Genotoxicity**

**In Vitro IMmortalization assay (IVIM)**

The IVIM assay was carried out using the principles described by Du et al., ^1^ with further modifications as reported in Modlich et al.,^2^. The LV.CHIM-p47 vector was the test item. The mock transduced cells receiving no viral vector served as a negative control, while the RSF91.GFPgPRE (RSF91) vector served as the positive control. Briefly, lineage marker negative bone marrow cells were isolated and stored at -80°C. The cells were then thawed out and pre-stimulated in StemSpan complete growth media (50 ng/ ml mSCF, 20 ng/ ml mIL3, 100 ng/ ml hFlt3-L and hIL11, all from Peprotech) for two days in 24 well plates. The transduction wells were coated with Retronectin and the vector was loaded first. The pre-stimulated cells were then transferred into the transduction wells for two rounds of transduction. Cells were counted, and transferred into Iscove’s Modified Dulbecco’s Medium (IMDM) (Biochrome) complete growth media for expansion, in 12 well plates. Three days later, cells were expanded further in 6 well plates. The cells transduced by GFP control vectors were analyzed by flow cytometry on days 4 and 15 after the second transduction. Cell counts were recorded on days 1, 4, 6, 8, 11 and 15 after the second round of transduction. Samples were taken for DNA isolation on days 4 and 15, and for RNA isolation only on day 15.

For clonal scoring, fifteen days after the second transduction, cells were seeded at 100 cells per well in 96 well plates. Between 28 to 31 days after the second transduction, cells were microscopically evaluated for clonal outgrowth, in conjunction with MTT assay, to determine clonal scoring. Each well of the 96 well plate is assigned one of the four categories (C1 to C4) according to a) the visible intensity of clonal outgrowth microscopically and b) the metabolic activity of the cells upon exposure to MTT (3-(4,5-Dimethylthiazol-2-yl)-2,5-diphenyltetrazolium bromide) and recording the fluorescence absorbance of the cells at 540 nm. The categories are as follow: C1, wells with a robust outgrowth; C2, wells with less outgrowth than in C1; C3, wells with some outgrowth that can be eventually assigned to a C2 group following the MTT test if samples scored > than the Q1-quantification threshold; C4, wells with no cells. The frequency of mutants with a replating phenotype is calculated according to Poisson statistics using the L-Calc software (Stem Cell Technologies, Vancouver, BC, Canada).

**In vivo**

**Mouse model and irradiation**

p47^phox-/-^mice (B6 [Cg]-Ncf1^m1J/J^) were originally provided by Dr Ulrich Siler, University of Zurich and bred in our facilities. Wild type C57BL/6 mice (from Charles River Laboratories, Netherlands) were used as donors for the control group. Procedures were approved by the UCL Biological Services Animal Welfare and Ethical Review Board, and licensed by the Home Office under the UK Animals (Scientific Procedures) Act 1986, Amendment Regulations 2012 (ASPA). Recipient p47^phox-/-^ mice were conditioned with 9 Gy (split dose) of irradiation; 6Gy and 3Gy administered at 24hrs and 5 hrs before tail vain injections. Transplanted mice were monitored daily during the first 2 weeks after transplantation and then monthly to record body weight. Mice were periodically bled to analyse DHR, VCN and total blood count. Mice showing weight loss $\geq$20% or signs of distress were humanely sacrificed following the NC3R recommendations.

**Murine Lin- isolation and culture**

Eight weeks old mice of mixed sex were sacrificed for lineage negative (Lin-) isolation. Lin- purification was performed with Mouse Lineage Cell Depletion Kit (Miltenyi Biotec, CA). Lineage negative cells were cultured in Stem Spam media (STEMCELL Technologies, Vancouver, Canada), 1% penicillin/streptomycin supplemented with mSCF (100ng/ml), mFLt3 (100ng/ml) and hTPO (25ng/ml) (PeproTech, Rocky Hill, NY).

**Transduction and transplantation**

Transduction of the Lin- cells was performed with a low dose (MOI 50) and a high dose (MOI 300) of the “GMP-comparable” LV.CHIM-p47 vector; LV. p47low and LV. p47high, respectively. The MOI 300 dose was achieved by two consecutive (15hrs and 8hrs) transductions of MOI 150 each. Lin- from wild type C57BL/6 mice or from p47^phox-/-^ mice were used as positive and negative controls. Before transplantation, cells were washed and resuspended in 200µl of PBS (5x10^5^ cells per mouse) and intravenously injected into the lethally irradiated p47^phox-/-^ mice. A total of 32 mice (16 males and 16 females) were used in this experiment (n=8 per group).

**Dihydrorhodamine (DHR) assay**

The DHR test was performed on 50µl of blood or 1x10^6^ cells from bone marrow at termination. Cells were co-incubated with anti-mouse CD11b-APC (clone M1/70, Biolegend) and anti-mouse Gr1-Pecy7 (clone RB6865, Biolegend) antibodies. When using blood, erythrocytes were lysed with 1x red blood cell lysis buffer (RBC) (Invitrogen) for 10 min at room temperature and washed with PBS prior the antibody staining. After a washing step with PBS-gg, stained cells were incubated in 1ml with 2.9µM of dihydrorhodamine 123 (DHR; Sigma-Aldrich) in PBSgg (0.05% gelatine, 0.09% D-glucose) containing 150U/ml of catalase (Sigma-Aldrich) for 15 min at 37°C. Samples were then divided in two (500µl each) of which one of them was subsequently activated with 1µg/ml PMA (Sigma-Aldrich) and incubated for another 15 min at 37°C. Cells were put on ice and acquired in CyAn^TM^ ADP (Beckman Coulter) flow cytometer within 30 min.

**Genomic DNA extraction**

gDNA from blood samples and/or haematopoietic organs (bone marrow, blood, spleen, thymus) was extracted using the DNeasy Blood and Tissue Kit (QiAgen) following the manufacturer’s protocol. DNA concentration for each sample was determined by Nanodrop (Thermo Scientific, USA).

**Vector copy number determined by ddPCR**

Average ector copy number/cell (VCN) was analysed by droplet digital PCR. DNA samples were diluted in water to 10ng/µl. Reactions were performed in a final volume of 22 µl, containing 1x ddPCR Supermix for probes (BioRad, Hercules, CA), 0.8µl of each primer (10µM) and 0.4µl of each probe (10µM), 2µl of DNA per sample and 5µl of nuclease free water. 20,000 droplets were generated using the automated droplet generator (BioRad) before amplification with C1000 Touch™ Thermal Cycler; 96–Deep Well Reaction Module (BioRad). Positives and negatives droplets were quantified using the QX200 Droplet reader (BioRad) and analyzed by Poisson statistics using QuantaSoft Software (BioRad). Concentration was provided by Quantasoft software as copies of the relevant gene per µl (copies/µl).

VCN was calculated as the ratio between the target gene concentration and the reference gene concentration, times number of copies of the reference gene per cell (x2): Ψ copies/mTitin copies *2. The Limit of detection (LOD) for this assay is defined as the lowest detectable concentration that could be determined with a certain level of confidence by the QX200; LOD > 0.001 copies per µl. The limit of quantification (LOQ) was defined as the lowest concentration for which the method provides results with an acceptable certainty; LOQ < 4 copies of mTitin per µl when calculating vector copy number.

Primers and probes used in this experiment are listed below:

| HIV-psi Forward | 5’ CAG GAC TCG GCT TGC TGA AG 3’ |
| --- | --- |
| HIV-psi Reverse | 5’ TCC CCC GCT TAA TAC TGA CG 3’ |
| HIV-psi Probe | 5’ FAM-CGC ACG GCA AGA GGC GAG G ZEN-3’ |
| TITIN Forward | 5’ ACCGAGAGAGGTGGTATTGA 3’ |
| TITIN Reverse | 5’ AGGATGCCTCCTGCTTAGA 3’ |
| TITIN Probe | 5’ HEX- AGCGTCTCGTCTCAGTCAGTCCAA -ZEN 3’ |

**Blood parameters**

In order to measure the white cell (WBC), red blood cell (RBC), platelet counts and haemoglobin levels, blood samples were acquired in the Sysmex analyser after a 1:4 dilution in PBS (XE-500).

**Histopathology**

Formalin fixed samples of thymus, spleen and tibia from 39 mice were processed by Propath Laboratories (UK). Slides from each of the sample were dewaxed in xylene, hydrated through a series of graded alcohols and rinsed in running tap water. The slides were then stained in Mayer’s haematoxylin before counter staining with aqueous eosin. Stained slides were then scanned on the Hamamatsu Nanozoomer 2.0 HT at 20x magnification to produce whole slide digital scans. Each of the 78 digital scans were evaluated by the study pathologist using the NDP.view2 viewing software. Each H&E section was thoroughly examined histologically, and lesions observed were recorded in an Excel spreadsheet, their severity graded (minimal, mild, moderate, or severe). Their distribution was also characterized (focal, multifocal, focally extensive or diffuse), as well as, their localization. Examination was performed in a blinded manner without knowledge of animal age, sex, strain, and/or treatment. The histopathological evaluation was performed at Sciempath Bio (Lillois, Belgium). Findings were reviewed by a second pathologist. A detailed description of the evaluation can be found in Supplementary Information.

**Flow Cytometry**

Lineage distribution

For lineage staining, single cell suspensions were prepared from Peripheral blood, Bone Marrow, Lymph Node, Spleen and Thymus. The Lineage stain was aimed at identifying three main population of cells namely – B-cells, T-cells, myeloid cells. For each tissue, 1x10^5^ cells were resuspended in 50µl MACS buffer and transferred into a (V-shaped) 96 well plates for staining. Before staining, samples were incubated with 1µl BD FC block (BD Pharmingen, Oxford, UK) for 5 minutes. Then cells were incubated at 4°C for 30 minutes with the following antibodies: mGR1-Pecy-7 (Biolegend, #108416); mCD11b-FitC (Biolegend, #101206); mCD3-Percp (Biolegend, #100326); mB220-Pacificblue, #103227); mCD45.2-APC (Biolegend, #109814).

Cells were washed with 200µl PBS, spun down at 400 G for 5 minutes, and the pellet was resuspended in 200µl of MACS buffer containing 1µg/ml DAPI (Biolegend, UK) and acquired using a BD LSRII cytometer.

p47^phox^ staining

For p47^phox^ detection, cells were first stained with a fixable live/dead marker (Zombie yellow-

Cat #423104, Biolegend, UK) and BD-Fc Block in PBS. The surface marker staining was done with anti-mouse GR1, CD11b, CD3 and B220 as described above. Cells were then fixed and permeabilised using Reagent A and Reagent B respectively of the NordicMUbio Fix & Perm kit (Cat # GAS-002), according to the manufacturer’s instructions. During the permeabilization step, 2µl of p47^phox^ antibody (BD transduction labs – cat # 624076) was added to reagent B, and was incubated for 15 minutes. The cells were then spun down, the pellet resuspended in 200ul MACS buffer and acquired using a BD LSRII cytometer. Post-acquisition data were analysed using FlowJo software (TreeStar, Ashland, OR, USA).

**Biodistribution**

**CD34+ HSPC and viral transduction**

Healthy donor CD34+ cells were obtained from a G-CSF mobilised apheresis (ALLCells) using the CliniMACS Prodigy system. Transduction of CD34+ cells was performed in X-Vivo20 media (Lonza, Switzerland), 1% HAS, supplemented with hFLt3 (100ng/ml), hSCF (100ng/ml), hTPO (20ng/ml) and hIL3 (20ng/ml) with the GMP-comparable LV. CHIM-p47 vector in the presence of Protamine Sulfate (4µg/ml). Transduction was performed in two steps 12hrs apart, each hit of MOI 100.

**Transplantation into NSG mice**

A total of 40 mice (20 males and 20 females) NSG mice (NOD.Cg-*Prkdc^scid^ Il2rg^tm1Wjl^*/SzJ) were purchased from Charles River laboratories. Animals were sub-lethally irradiated (2.5Gy) and 24hrs later were transplanted with 5x10^5^ untransduced (UN) or LV. CHIM-p47-transduced (LV. p47) CD34+ cells. Transplanted mice (10 males and 10 females for each group) were observed daily during the first 2 weeks after the procedure to monitor body weight and report clinical signs of illness. Eleven weeks post transplantation, organs (bone marrow, peripheral blood, spleen, thymus, lymph nodes, lung, liver, heart, brain, kidney, gonads and muscle) were collected for analysis after perfusion in PBS. Haematopoietic organs were analysed by FACS for expression of hCD45+ cells and by ddPCR for VCN and human engraftment (hAlbumin vs. mTitin). Non-haematopoietic organs were analysed by ddPCR for VCN and human engraftment (hAlbumin vs. mTitin).

**Genomic DNA (gDNA) extraction**

gDNA from blood samples and/or haematopoietic organs (bone marrow, blood, spleen, thymus) was extracted using the DNeasy Blood and Tissue Kit (QiAgen) following the manufacturer’s protocol. Hard tissues from non-haematopoietic organs (liver, heart, lung, kidney, brain, muscle and gonads) were homogenised prior to gDNA extraction with DNAeasy Blood and Tissue Kit (QiAgen). Homogenisation was performed in Precellys’ tubes containing ceramic beads and 200-300µl homogenisation buffer (D-PBS+1% Triton X-100) depending of the size of the organ, and 20 µl of proteinase K. In Precellys homogeniser, program 1 was run (2 cycles at 5,700 rpm, 30 sec each with intervals of 10 secs).
After centrifugation (13000 rpm for 1 min), supernatant was transferred into a clean Eppendorf tube and processed further for DNA extraction. DNA concentration for each sample was determined by Nanodrop (Thermo Scientific, USA).

**VCN by ddPCR**

Average vector copy number/cell (VCN) was calculated as previously described in the Genotoxicity section. The set of primers and probes used are listed below: Ψ copies/hAlb copies *2.

| HIV-psi Forward | 5’ CAG GAC TCG GCT TGC TGA AG 3’ |
| --- | --- |
| HIV-psi Reverse | 5’ TCC CCC GCT TAA TAC TGA CG 3’ |
| HIV-psi Probe | 5’ FAM-CGC ACG GCA AGA GGC GAG G ZEN-3’ |
| Albumin Forward | 5’ GCT GCT ATC TCT TGT GGG CTG T 3’ |
| Albumin Reverse | 5’ ACT CAT GGG AGC TGC TGG TTC 3’ |
| Albumin Probe | 5’ HEX-CCT GTC ATG CCC ACA CAA ATC TCT CC-ZEN 3’ |

The LOD was set as before at > 0.001 copies per µl, and the LOQ was fixed at < 4 copies of human albumin per µl for vector copy number. According to this, VCN could be assessed in the BM and spleen of all LV. p47 mice, in the PB of 3 males and 3 females, in the thymus of 4 females and 5 males, in the liver of 7 females and 3 males, in the kidneys of 7 females and 10 males, in the lung of 9 females and 7 males, in the muscle of 3 females and 3 males and in the gonads of 1 female.

**Human engraftment analysis by ddPCR**

Percentage of human engraftment was evaluated by dividing the human albumin genome over total genome (murine titin + human albumin). The LOQ was set at < 4 copies of murine titin per µl when calculating copies of albumin over titin. For non-haematopoietic organs, we increased the amount of DNA used in order to have generally values of titin per well> 5000. The set of primers and probes used for the detection of human engraftment used are as follows:

| Albumin Forward | 5’ GCTGCTATCTCTTGTGGGCTGT 3’ |
| --- | --- |
| Albumin Reverse | 5' AGCTGGGATTCACTCAGTTTG 3' |
| Albumin Probe | 5' FAM- CCTGTCATGCCCACACAAATCTCTCC -ZEN 3' |
| TITIN Forward | 5’ ACCGAGAGAGGTGGTATTGA 3’ |
| TITIN Reverse | 5’ AGGATGCCTCCTGCTTAGA 3’ |
| TITIN Probe | 5’ HEX- AGCGTCTCGTCTCAGTCAGTCCAA -ZEN 3’ |

**Flow cytometry**

Detection of engrafted cells. Human cell engraftment was assessed by staining PB, BM, spleen or thymus cell suspension with the anti-CD45RA-PE antibody (Tonbo Bioscience, catalog #65-0458-T100). As isotype control mouse IgG1-Kappa-PE was used (Ref n: 12-4714-42; Lot n: E11419-1632). Cells were recorded on the BD LSR II flow cytometer (BD Biosciences, Oxford, UK) using BD FACS Diva software, and data were analysed using FlowJo software (TreeStar, Ashland, OR, USA). The LOQ was set at >1% CD45+ cells.

Lineage distribution in NSG mice. Cell suspensions from BM, spleen, thymus and peripheral blood (approximately 2x10^5^ cells) were co-stained in 50µl MACS buffer with 0.5µl of each of the following antibodies: CD45-Pe (BD Bioscience, catalogue #555483), CD13-PeCy (BD Bioscience, catalogue #338439), CD3-FitC (BD Bioscience, catalogue #345763) and CD19-APC (BD Bioscience, catalogue #55415). Prior to acquisition, samples were stained with 2µl of the viability dye DAPI. Samples were analysed on BD LSR II flow cytometer (BD Biosciences, Oxford, UK) using BD FACS Diva software, and data were analysed using FlowJo software (TreeStar, Ashland, OR, USA).

**Lentiviral transduction of CD34 + cells with transduction enhancers**

Peripheral blood mobilised CD34+ cells from healthy donors and from one p47^phox^ CGD patient were obtained after written informed consent under the auspices of National Institute of Allergy and Infectious Diseases (NIAID) Institutional Review Board-approved protocols 05-I-0213 and 94-I-0073 (HLM PI for protocols). The conduct of these studies conformed to the Declaration of Helsinki protocols and all United States federal regulations required for protection of human subjects.

Fresh or cryopreserved CD34+ cells, after thawing, were cultured in 1% human serum albumin/X-Vivo 10 with SCF, FLT3-lig, and TPO at 100ng/ml for 48 hr before transduction with different doses of vector in the presence of 4μg/ml of Protamine Sulphate and 1mg/ml of LentiBOOST (GMP grade Kolliphor P338, BASF). After transduction, cells were cultured in myeloid differentiation medium, Iscove’s Modified Dulbecco’s Medium (Gibco; Thermo Fisher Scientific), supplemented with 20% fetal bovine serum (Atlanta Biologicals; R&D Systems; Minneapolis, MN) and hG-CSF (50ng/ml; Peprotech) for 14 days to assess VCN and/or NADPH oxidase activity and p47^phox^ expression as previously described. 1x10^6^ CD34+ cells were transplanted into NOD.Cg-Prkdcscid Il2rgtm1Wjl/SzJ (NSG) (The Jackson Laboratory; Bar Harbor, ME) mice, that were conditioned with an intraperitoneal injection of 20 mg/kg busulfan approximately 24 hrs before transplant (3 mice received untreated p47CGD cells, 3 mice received untreated healthy donor cells, 4 mice received p47CGD cells that had been transduced with the LV.CHIM-p47 vector). Xenotransplant studies were approved by the NIAID Institutional Animal Care and Use Committee under the animal use protocol ASP LCIM 1E. The conduct of the experiments conformed to Association for Assessment and Accreditation of Laboratory Animal Care International guidelines and all U.S. federal regulations governing the protection of research animals (HLM PI for the protocol).

**Statistical analysis**

For comparison among two groups, we used the non-parametric Mann-Whitney test. For comparisons among more than two groups we used the analysis of variance with Sidak’s or Tukey’s multiple comparisons. *P-*values < 0.05 were considered significant. All tests were performed using GraphPad Prism version 8. Specifics of each statistical test used can be found in figure legends.

**SUPPLEMENTARY INFORMATION**

**Histopathological analysis of thymus, spleen and bone marrow**

For a better assessment of the toxicological effects of our lentiviral gene therapy in mice, we evaluated histologically haematopoietic organs of treated and control animals in a blind manner. The histopathological evaluation gives an overview of the location of the lesions at cellular or structural levels ^3^.

Samples from a total of 39 animals were sent for histopathology analysis to Propath, of which 15 animals received p47^phox-/-^ Lin^-^ cells transduced with a high (LV. p47 high) or low dose (LV. p47 low) of the LV.CHIM-p47 vector (Group A+B), 7 animals received non-transduced p47^phox-/-^ (Mock) or wild type (HCT) cells for a total of 14 animals (Group C and D, respectively). We included 10 animals as further controls (four untreated p47^phox-/-^ and six C57BL/6 mice; Group E and F, respectively).

Groups of animals and animal ID

| **Group** | **Animal ID** |  | **Group** | **Animal ID** |  | **Group** | **Animal ID** |
| --- | --- | --- | --- | --- | --- | --- | --- |
| Group A | 806548 |  | Group C | 809604 |  | Group F | 823786 |
|  | 807312 |  |  | 803928 |  |  | 823787 |
|  | 809602 |  |  | 812780 |  |  | 823788 |
|  | 807315 |  |  | 806546 |  |  | 823781 |
|  | 812783 |  |  | 748306 |  |  | 823782 |
|  | 806542 |  |  | 806540 |  |  | 823783 |
|  | 748297 |  |  | 748298 |  | **WT** |  |
| **LV. p47 high** | **MOI 300** |  | **MOCK** |  |  |  |  |
|  |  |  |  |  |  |  |  |
|  |  |  |  |  |  |  |  |
| **Group** | **Animal ID** |  | **Group** | **Animal ID** |  | **Group** | **Animal ID** |
| Group B | 806547 |  | Group D | 807311 |  | Group E | 748304 |
|  | 807310 |  |  | 809603 |  |  | 803929 |
|  | 748303 |  |  | 807313 |  |  | 803930 |
|  | 807314 |  |  | 812782 |  |  | 809601 |
|  | 812779 |  |  | 806544 |  | **KO** |  |
|  | 812781 |  |  | 748305 |  |  |  |
|  | 806545 |  |  | 748299 |  |  |  |
|  | 806541 |  | **HCT** |  |  |  |  |
| **LV.p47 low** | **MOI 50** |  |  |  |  |  |  |
|  |  |  |  |  |  |  |  |

All thymus sections examined presented normal structure, however most of the animals had hypercellularity of mature lymphocytes in the cortex, a feature commonly seen in young and active organs ^4^.

The histological cuts of the spleen showed in most of the animals a focal high cellular density in the periarterial lymphatic sheath (PALS), a compartment of the white pulp with mostly T cells. This focal accumulation of lymphocytes and macrophages has been previously reported as spontaneous lesion in other animal models like F334 rats ^5^. No difference between groups was observed in the white pulp of the spleen. When looking at the red pulp, two animals belonging to group E (KO) and C (Mock) (803930 and 803928 respectively), presented a mild to severe macrophage infiltration clustered forming a multifocal granuloma-like lesion, typical symptom of CGD disease. One mouse from group B (812781) presented a mild increased number on the megakaryocytes.

The bone marrow of most of the mice analysed presented a mild diffuse hypercellularity related to a decrease in adipocytes ^5^. All lineages were present in the increased cellular density, finding no differences between groups. One mouse belonging to group C (806540) presented macrophages infiltration. Below a summary of histopathology findings for each animal.

| **Group** | **Animal ID** | **Organ** | **Finding** | **Grade** | **Distribution** | **Modifier** |
| --- | --- | --- | --- | --- | --- | --- |
| **A+B** | 748303 | Thymus | unremarkable | - | - | - |
|  |  | Spleen | unremarkable | - | - | - |
|  |  | Bone marrow | unremarkable | - | - | - |
|  | 806545 | Thymus | unremarkable | - | - | - |
|  |  | Spleen | unremarkable | - | - | - |
|  |  | Bone marrow | cellularity increased | mild | diffuse | granulocytic/myeloid |
|  | 806547 | Thymus | unremarkable | - | - | - |
|  |  | Spleen | unremarkable | - | - | - |
|  |  | Bone marrow | cellularity increased | minimal | diffuse | granulocytic/myeloid |
|  | 806548 | Thymus | unremarkable | - | - | - |
|  |  | Spleen | unremarkable | - | - | - |
|  |  | Bone marrow | cellularity increased | mild | diffuse | granulocytic/myeloid |
|  | 806541 | Thymus | unremarkable | - | - | - |
|  |  | Spleen | unremarkable | - | - | - |
|  |  | Bone marrow | cellularity increased | minimal | diffuse | granulocytic/myeloid |
|  | 806542 | Thymus | unremarkable | - | - | - |
|  |  | Spleen | unremarkable | - | - | - |
|  |  | Bone marrow | cellularity increased | mild | diffuse | granulocytic/myeloid |
|  | 807310 | Thymus | unremarkable | - | - | - |
|  |  | Spleen | unremarkable | - | - | - |
|  |  | Bone marrow | cellularity increased | minimal | diffuse | granulocytic/myeloid |
|  | 807312 | Thymus | unremarkable | - | - | - |
|  |  | Spleen | unremarkable | - | - | - |
|  |  | Bone marrow | cellularity increased | mild | diffuse | granulocytic/myeloid |
|  | 809602 | Thymus | unremarkable | - | - | - |
|  |  | Spleen | unremarkable | - | - | - |
|  |  | Bone marrow | cellularity increased | mild | diffuse | granulocytic/myeloid |
|  | 748297 | Thymus | unremarkable | - | - | - |
|  |  | Spleen | unremarkable | - | - | - |
|  |  | Bone marrow | unremarkable | - | - | - |
|  | 807314 | Thymus | unremarkable | - | - | - |
|  |  | Spleen | unremarkable | - | - | - |
|  |  | Bone marrow | cellularity increased | minimal | diffuse | granulocytic/myeloid |
|  | 807315 | Thymus | unremarkable | - | - |  |
|  |  | Spleen | unremarkable | - | - |  |
|  |  | Bone marrow | cellularity increased | minimal | diffuse | granulocytic/myeloid |
|  | 812779 | Thymus | unremarkable | - | - | - |
|  |  | Spleen | unremarkable | - | - | - |
|  |  | Bone marrow | unremarkable | - | - | - |
|  | 812781 | Thymus | unremarkable | - | - | - |
|  |  | Spleen | megakaryocytes, number increased | mild | multifocal | - |
|  |  | Bone marrow | unremarkable | - | - | - |
|  | 812783 | Thymus | unremarkable | - | - | - |
|  |  | Spleen | unremarkable | - | - | - |
|  |  | Bone marrow | cellularity increased | minimal | diffuse | granulocytic/myeloid |

| **Group** | **Animal ID** | **Organ** | **Finding** | **Grade** | **Distribution** | **Modifier** |
| --- | --- | --- | --- | --- | --- | --- |
| **C** | 812780 | Thymus | unremarkable | - | - | - |
|  |  | Spleen | unremarkable | - | - | - |
|  |  | Bone marrow | unremarkable | - | - | - |
|  | 748298 | Thymus | unremarkable | - | - | - |
|  |  | Spleen | unremarkable | - | - | - |
|  |  | Bone marrow | unremarkable | - | - | - |
|  | 809604 | Thymus | unremarkable | - | - | - |
|  |  | Spleen | unremarkable | - | - | - |
|  |  | Bone marrow | cellularity increased | mild | diffuse | granulocytic/myeloid |
|  | 806540 | Thymus | unremarkable | - | - | - |
|  |  | Spleen | unremarkable | - | - | - |
|  |  | Bone marrow | macrophage infiltration | mild | multifocal | - |
|  |  | Bone marrow | cellularity increased | mild | diffuse | granulocytic/myeloid |
|  | 806546 | Thymus | unremarkable | - | - | - |
|  |  | Spleen | unremarkable | - | - | - |
|  |  | Bone marrow | cellularity increased | mild | diffuse | granulocytic/myeloid |
|  | 748306 | Thymus | unremarkable | - | - | - |
|  |  | Spleen | unremarkable | - | - | - |
|  |  | Bone marrow | no sample submitted for examination | - | - | - |
|  | 803928 | Thymus | unremarkable | - | - | - |
|  |  | Spleen | macrophage infiltration | severe | multifocal | red pulp |
|  |  | Bone marrow | cellularity increased | mild | diffuse | granulocytic/myeloid |
| **D** | 748305 | Thymus | unremarkable | - | - | - |
|  |  | Spleen | unremarkable | - | - | - |
|  |  | Bone marrow | cellularity increased | mild | diffuse | granulocytic/myeloid |
|  | 806544 | Thymus | unremarkable | - | - | - |
|  |  | Spleen | unremarkable | - | - | - |
|  |  | Bone marrow | cellularity increased | mild | diffuse | granulocytic/myeloid |
|  | 807311 | Thymus | unremarkable | - | - | - |
|  |  | Spleen | unremarkable | - | - | - |
|  |  | Bone marrow | cellularity increased | mild | diffuse | granulocytic/myeloid |
|  | 809603 | Thymus | unremarkable | - | - | - |
|  |  | Spleen | unremarkable | - | - | - |
|  |  | Bone marrow | cellularity increased | minimal | diffuse | granulocytic/myeloid |
|  | 748299 | Thymus | unremarkable | - | - | - |
|  |  | Spleen | unremarkable | - | - | - |
|  |  | Bone marrow | unremarkable | - | - | - |
|  | 807313 | Thymus | unremarkable | - | - | - |
|  |  | Spleen | unremarkable | - | - | - |
|  |  | Bone marrow | cellularity increased | mild | diffuse | granulocytic/myeloid |
|  | 812782 | Thymus | unremarkable | - | - | - |
|  |  | Spleen | unremarkable | - | - | - |
|  |  | Bone marrow | unremarkable | - | - | - |

| **Group** | **Animal ID** | **Organ** | **Finding** | **Grade** | **Distribution** | **Modifier** |
| --- | --- | --- | --- | --- | --- | --- |
| **F** | 823786 | Thymus | unremarkable | - | - | - |
|  |  | Spleen | unremarkable | - | - | - |
|  |  | Bone marrow | cellularity increased | minimal | diffuse | granulocytic/myeloid |
|  | 823787 | Thymus | unremarkable | - | - | - |
|  |  | Spleen | unremarkable | - | - | - |
|  |  | Bone marrow | cellularity increased | mild | diffuse | granulocytic/myeloid |
|  | 823788 | Thymus | unremarkable | - | - | - |
|  |  | Spleen | unremarkable | - | - | - |
|  |  | Bone marrow | cellularity increased | minimal | diffuse | granulocytic/myeloid |
|  | 823781 | Thymus | unremarkable | - | - | - |
|  |  | Spleen | unremarkable | - | - | - |
|  |  | Bone marrow | cellularity increased | mild | diffuse | granulocytic/myeloid |
|  | 823782 | Thymus | unremarkable | - | - | - |
|  |  | Spleen | unremarkable | - | - | - |
|  |  | Bone marrow | cellularity increased | mild | diffuse | granulocytic/myeloid |
|  | 823783 | Thymus | unremarkable | - | - | - |
|  |  | Spleen | unremarkable | - | - | - |
|  |  | Bone marrow | cellularity increased | mild | diffuse | granulocytic/myeloid |
| **E** | 748304 | Thymus | unremarkable | - | - | - |
|  |  | Spleen | macrophage infiltration | mild | multifocal | red pulp |
|  |  | Bone marrow | cellularity increased | mild | diffuse | granulocytic/myeloid |
|  | 803929 | Thymus | unremarkable | - | - | - |
|  |  | Spleen | unremarkable | - | - | - |
|  |  | Bone marrow | cellularity increased | minimal | diffuse | granulocytic/myeloid |
|  | 803930 | Thymus | unremarkable | - | - | - |
|  |  | Spleen | macrophage infiltration | moderate | multifocal | red pulp |
|  |  | Bone marrow | cellularity increased | mild | diffuse | granulocytic/myeloid |
|  | 809601 | Thymus | unremarkable | - | - | - |
|  |  | Spleen | unremarkable | - | - | - |
|  |  | Bone marrow | cellularity increased | mild | diffuse | granulocytic/myeloid |

The Histopathology phase of this study was conducted according to the study protocol in a facility compliant with the UK Principles of Good Laboratory Practice (The United Kingdom GLP Regulations 1999), Statutory Instrument No. 3106 as amended by the Good Laboratory Practice (Codification Amendment Etc) Regulations 2004 Statutory Instrument No. 994. These are in accordance with the Organisation for Economic Co-operation and Development (OECD) Principles of Good Laboratory Practice (1997) (ENV/MC/CHEM (98) 17) although no claim of compliance is made.

**1.** Du Y, Jenkins NA, Copeland NG: Insertional mutagenesis identifies genes that promote the immortalization/self-renewal of primary bone marrow progenitor cells. *Blood.* 2005;106(11):60a-60a.

**2.** Modlich U, Navarro S, Zychlinski D, et al.: Insertional transformation of hematopoietic cells by self-inactivating lentiviral and gammaretroviral vectors. *Mol Ther.* 2009;17(11):1919-1928.

**3.** Reagan WJ, Irizarry-Rovira A, Poitout-Belissent F, et al.: Best practices for evaluation of bone marrow in nonclinical toxicity studies. *Toxicol Pathol.* 2011;39(2):435-448.

**4.** Elmore SA: Enhanced histopathology of the thymus. *Toxicol Pathol.* 2006;34(5):656-665.

**5.** Elmore SA: Enhanced histopathology of the bone marrow. *Toxicol Pathol.* 2006;34(5):666-686.
