# Supplementary material for: Preclinical Optimization and Safety Studies of a New Lentiviral Gene Therapy for p47phox-Deficient Chronic Granulomatous Disease
Source: Hum Gene Ther. 2021 Sep 23;32(17-18):949–58. doi: 10.1089/hum.2020.276 (PMC8575060; doi:10.1089/hum.2020.276)
Supplement: Supplemental data [file Supp_TableS1.pdf]

Supplementary Tables and Figures

Supplementary Table 1. Details of VCN and GFP expression in samples from the IVIM assay

	Sample ID	Vector	c.MOI	VCN (day 4)	VCN (mean±SD)	eGFP (in % day 4)	eGFP (in % day 15)
190109	190109-4	RSF91	60	6.80		98.10%	98.10%
190109	190109-5	RSF91	60	7.31		99.20%	99.40%
190109	190109-6	RSF91	60	6.66		98.60%	99.90%
190206	190206-4	RSF91	60	7.02		97.60%	99.20%
190206	190206-5	RSF91	60	7.78	**7.50 ± 0.73**	97.80%	98.40%
190206	190206-6	RSF91	60	8.78		97.70%	95.90%
190220	190220-4	RSF91	60	7.29		97.60%	99.10%
190220	190220-5	RSF91	60	8.52		97.70%	97.40%
190220	190220-6	RSF91	60	7.38		97.70%	98.20%
190109	190109-10	LV.p47	400	7.80		-	
190109	190109-11	LV.p47	400	7.68		-	
190109	190109-12	LV.p47	400	7.61		-	
190109	190109-7	LV.p47	400	8.46		-	
190109	190109-8	LV.p47	400	6.71		-	
190109	190109-9	LV.p47	400	7.08		-	
190206	190206-10	LV.p47	400	7.06		-	
190206	190206-11	LV.p47	400	6.19	**7.56 ± 1.74**	-	
190206	190206-12	LV.p47	400	5.84		-	
190206	190206-7	LV.p47	400	9.68		-	
190206	190206-8	LV.p47	400	6.89		-	
190206	190206-9	LV.p47	400	9.86		-	
190220	190220-13	LV.p47	400	11.51		-	
190220	190220-14	LV.p47	400	6.16		-	
190220	190220-15	LV.p47	400	4.80		-	
