# Supplementary material for: Preclinical Optimization and Safety Studies of a New Lentiviral Gene Therapy for p47phox-Deficient Chronic Granulomatous Disease
Source: Hum Gene Ther. 2021 Sep 23;32(17-18):949–58. doi: 10.1089/hum.2020.276 (PMC8575060; doi:10.1089/hum.2020.276)
Supplement: Supplemental data [file Supp_FigS2.pdf]

Supplementary Figure 2

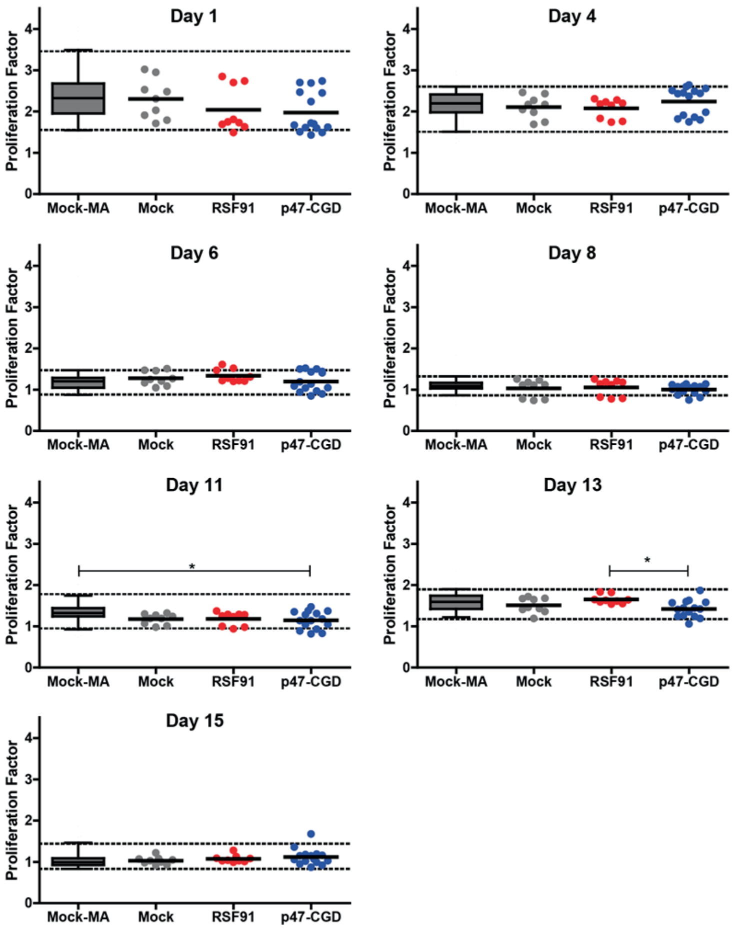

Supplementary Figure 2. Proliferation rate of samples at different time points (days) in the IVIM assay. Dotted lines mark 5% and 95% percentile values of meta-analysis data from 82 Mock controls. Differences in the proliferation behaviour relative to the current Mock samples were analysed by Kruskal-Wallis test with Dunn's correction (*$P < 0.05$). Bars indicate means.
