# Supplementary material for: Preclinical Optimization and Safety Studies of a New Lentiviral Gene Therapy for p47phox-Deficient Chronic Granulomatous Disease
Source: Hum Gene Ther. 2021 Sep 23;32(17-18):949–58. doi: 10.1089/hum.2020.276 (PMC8575060; doi:10.1089/hum.2020.276)
Supplement: Supplemental data [file Supp_TableS2.pdf]

Supplementary Table 2. VCN and DHR values in the peripheral blood of gene therapy-treated mice at different time points.

Average vector copy number/cell (VCN)

mouse ID	807310	807314	806545	806541	806547	748302	812779	812781	807315	803927	807312	806542	806548	809602	812783	748297
code	#1	#2	#3	#4	#5	#6	#7	#8	#9	#10	#11	#12	#13	#14	#15	#16
1M	2.2	3.6	3.3	3.4	3.5	3.9	3.9	4.2	5.9	0.5	6.5	6.5	ND	6.4	7	5
3M	2.7	3.8	2.8	3.7	3.4	2.6	3.5	3.5	6.4	0	5.9	5	6.4	5.4	6.5	5.6
6M	2.4	4.3	3.3	3.5	3.2	2.1	3.7	4.3	9.1		6.9	5.1	3.3	4.9	6.9	4.5

% of DHR out of granulocytes

mouse ID	807310	807314	806545	806541	806547	748302	812779	812781	807315	803927	807312	806542	806548	809602	812783	748297
code	#1	#2	#3	#4	#5	#6	#7	#8	#9	#10	#11	#12	#13	#14	#15	#16
1M	34.4	64.1	61.1	50.4	58.8	55.7	71.1	64	54	2	56.2	60.8	69.2	63.3	64.8	47
3M	24.4	68.3	63.7	77.6	70.6	62.3	70.5	ND	42.1	1.2	59	66.3	41.6	61.3	80	54
5M	17.2	68.3	45.5	68.7	74	57.3	64	56.2	54.1		56.2	58.9	31	63.3	74	42.1
6M	18.6	72.4	53.7	81.8	78.1	65.7	66.1	45.8	ND		58.1	61.4	37.9	60.8	71.4	54.1
