# Supplementary material for: Preclinical Optimization and Safety Studies of a New Lentiviral Gene Therapy for p47phox-Deficient Chronic Granulomatous Disease
Source: Hum Gene Ther. 2021 Sep 23;32(17-18):949–58. doi: 10.1089/hum.2020.276 (PMC8575060; doi:10.1089/hum.2020.276)
Supplement: Supplemental data [file Supp_FigS3.pdf]

Supplementary Figure 3

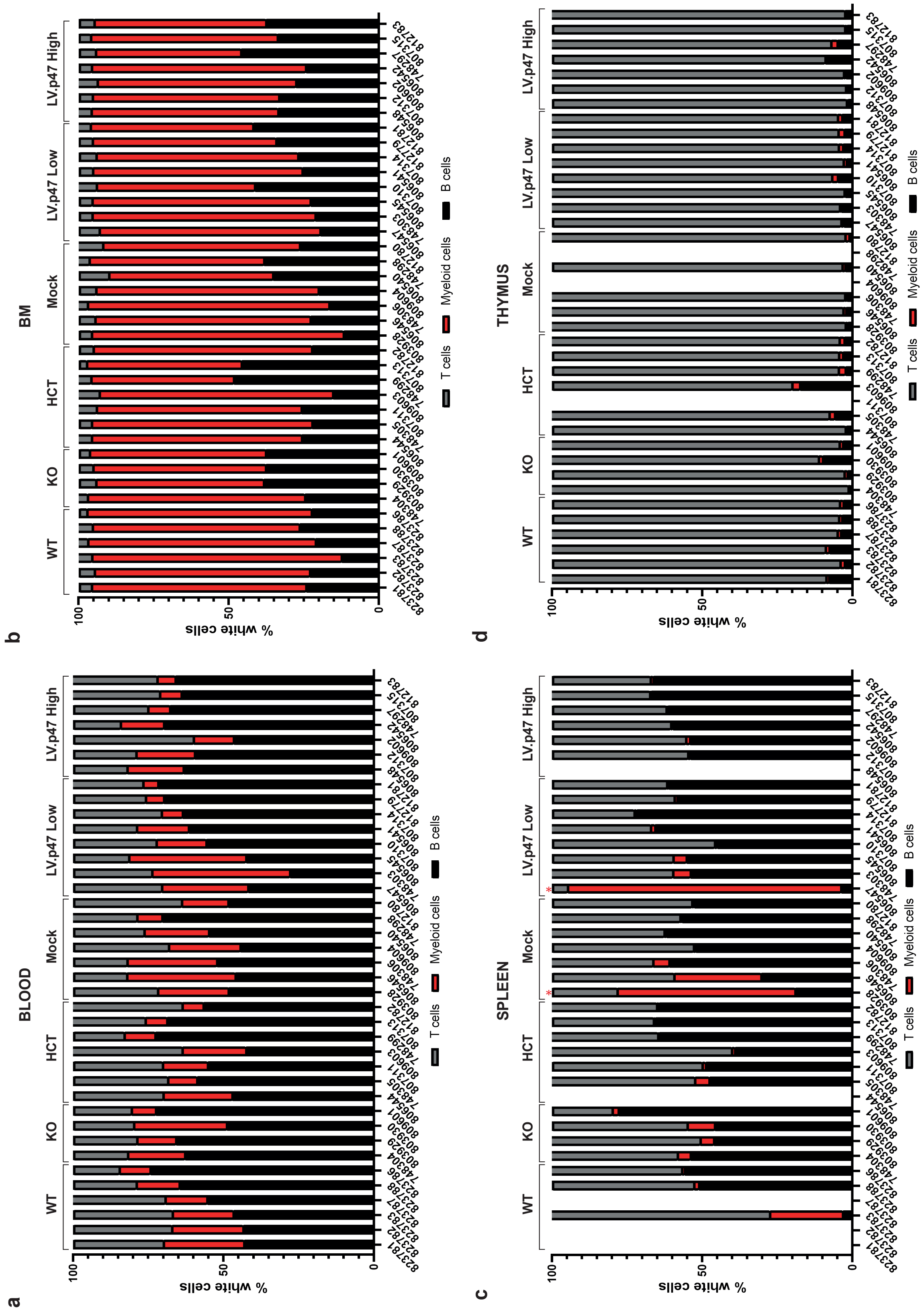

Supplementary Figure 3. Genotoxicity study in p47$^{phox\ -/-}$ mice: lineage representation in haematopoietic organs. Flow cytometric analysis of T cells (CD3+), B cells (B220+) and myeloid cells (CD11b+) in PB, BM , spleen or thymus of transplanted animals. 100% is given by the sum of B, T and myeloid cells percentages. Samples with less than 1000 events in the live (DAPI-ve) gate were discarded. Red astericks show samples of dubious technical validity due to the presence of > 90% dead events.
