# Supplementary material for: Preclinical Optimization and Safety Studies of a New Lentiviral Gene Therapy for p47phox-Deficient Chronic Granulomatous Disease
Source: Hum Gene Ther. 2021 Sep 23;32(17-18):949–58. doi: 10.1089/hum.2020.276 (PMC8575060; doi:10.1089/hum.2020.276)
Supplement: Supplemental data [file Supp_FigS4.pdf]

Supplementary Figure 4

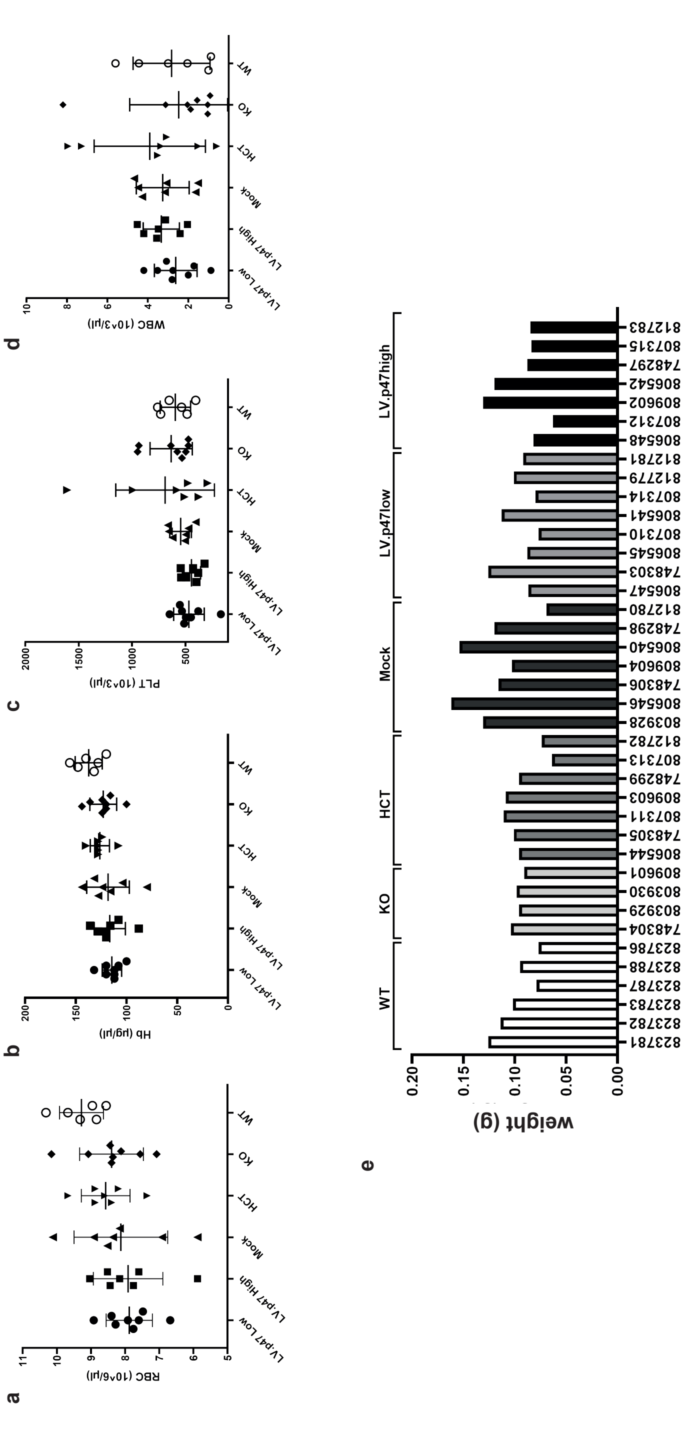

Supplementary Figure 4. Genotoxicity study in p47$^{phox -/-}$ mice: blood parameters and spleen weight of individual mice. Platelet (a), while blood cell (b), red blood cell (c) counts and (d) haemoglobin (µg/ml) in the peripheral blood of experimental mice. Data are mean ± SD; one-way ANOVA with by Tukey's multiple comparison; ns. e) spleen weight (g).
