# Supplementary material for: Preclinical Optimization and Safety Studies of a New Lentiviral Gene Therapy for p47phox-Deficient Chronic Granulomatous Disease
Source: Hum Gene Ther. 2021 Sep 23;32(17-18):949–58. doi: 10.1089/hum.2020.276 (PMC8575060; doi:10.1089/hum.2020.276)
Supplement: Supplemental data [file Supp_FigS5.pdf]

Supplementary Figure 5

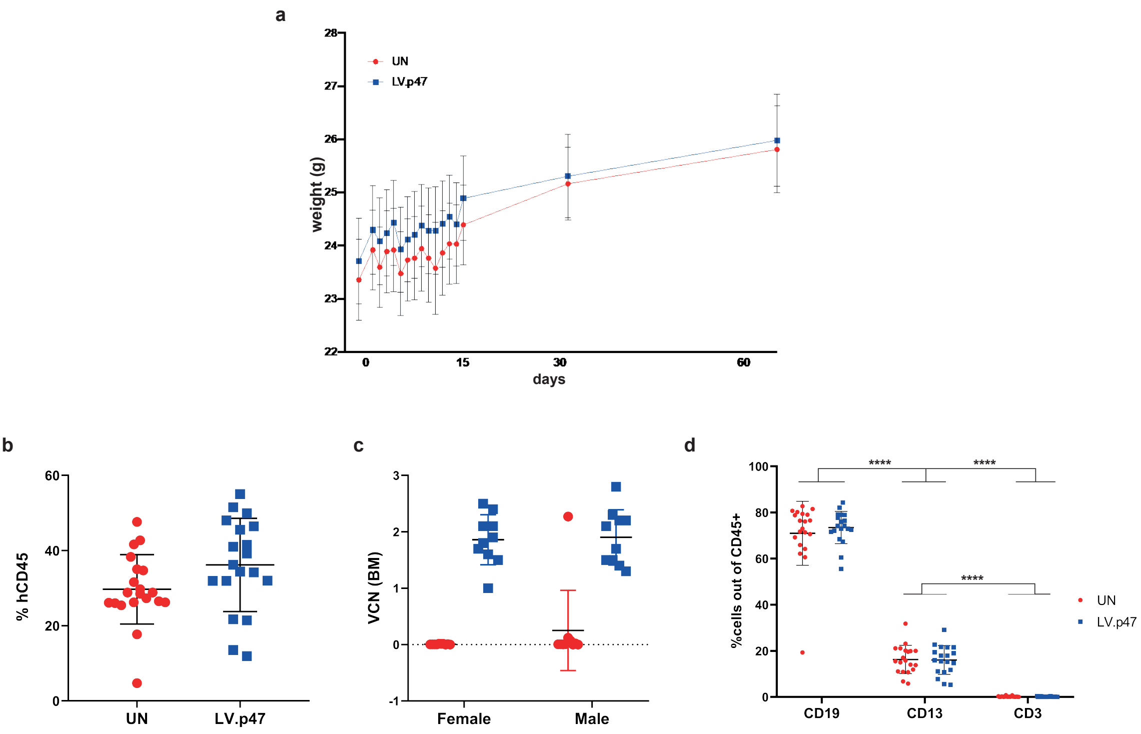

Supplementary Figure 5. Biodistribution of transduced CD34+ cells into NSG mice. a) Monitoring of body weight overtime in NSG mice transplanted with untransduced (UN) or LV.CHIM-p47-transduced (LV. p47) human CD34+ cells. b) Percentage of CD45+ cells in the bone marrow of NSG mice transplanted with untransduced (UN) or gene therapy-treated (LV. p47) CD34+ cells. Data are mean ±SD, n=20. Student's t test; ns: not significant c) VCN in females and males LV.47 mice. d) Lineage representation in bone marrow of UN (n=20) and LV. p47 mice (n=20). Data are mean ± SD; two-way ANOVA with Sidak's correction. ****p<0.001.
